# Supplementary material for: Acetylation of BMAL1 by TIP60 controls BRD4-P-TEFb recruitment to circadian promoters
Source: eLife. 2019 Jul 11;8:e43235. doi: 10.7554/eLife.43235 (PMC6650244; doi:10.7554/eLife.43235)
Supplement: Supplementary file 1. [file elife-43235-supp1.docx]

**Supplementary file 1.** **Two-way ANOVA statistical analysis**

|  |  | two-way ANOVA main effects | | | Bonferroni post-test |
| --- | --- | --- | --- | --- | --- |
| Figure | Gene | Time (p) | Genotype (p) | Interaction (p) | p-values below 0.05 are shown |
| 2C | *Dbp* | < 0.0001 | < 0.0001 | < 0.0001 | Clone 1/2/3: 24/28/32/36/40/44: < 0.001 |
| Figure 2 – figure supplement 1F | *Per1* | < 0.0001 | < 0.0001 | < 0.0001 | Clone 1: 24/28/32/36/44: < 0.001; 40 < 0.01  Clone 2: 24/28/44: < 0.001; 32 < 0.05  Clone 3: 24/28/40/44: < 0.001; 32/36: < 0.05 |
|  | *Nr1d1* | < 0.0001 | < 0.0001 | < 0.0001 | Clone 1: 24/28/40/44: < 0.001; 32 < 0.01  Clone 2: 24/28/32/36/40/44: < 0.001  Clone 3: 24/28/32/36: < 0.001 |
| 3E | *Dbp* | < 0.0001 | < 0.0001 | < 0.0001 | 24/28/32/36/40/44: < 0.0001 |
| 5D | *Per1* | < 0.0001 | < 0.0001 | < 0.0001 | CT 0/24: < 0.01; CT 6: < 0.0001 |
|  | *Bmal1* | 0.0002 | 0.0005 | 0.2175 | CT 18: < 0.01 |
|  | *Dbp* | < 0.0001 | 0.0002 | 0.0006 | CT 6: < 0.0001; CT12: < 0.05 |
| 5E | *Dbp* | < 0.0001 | < 0.0001 | < 0.0001 | 24/36/40: < 0.001; 28/32/44: < 0.0001 |
|  | *Nr1d1* | < 0.0001 | 0.0012 | < 0.0001 | 24: < 0.001; 28: < 0.0001 |
|  | *Per1* | 0.0002 | < 0.0001 | 0.0002 | 24/28/32/36/40/44: < 0.0001 |
|  | *Per2* | < 0.0001 | < 0.0001 | < 0.0001 | 24/28/32/36/44: < 0.0001; 40: < 0.01 |
|  | *Bmal1* | < 0.0001 | < 0.0001 | 0.0007 | 36/40: < 0.0001; 44: <0.01 |
|  | *Gapdh* | 0.2918 | 0.0079 | 0.5568 | - |
| Figure | Gene | Time (p) | Treatment (p) | Interaction (p) | p-values below 0.05 are shown |
| 1C | *Dbp* | < 0.0001 | < 0.0001 | < 0.0001 | 24/28/32/36/40/44: < 0.001 |
