# Supplementary material for: Acetylation of BMAL1 by TIP60 controls BRD4-P-TEFb recruitment to circadian promoters
Source: eLife. 2019 Jul 11;8:e43235. doi: 10.7554/eLife.43235 (PMC6650244; doi:10.7554/eLife.43235)
Supplement: Supplementary file 2. [file elife-43235-supp2.docx]

**Supplementary file 2. Primer Sequences**

| Tip60 Genotyping |  |
| --- | --- |
| P1 | GGAAGGTTCAAAATTCCAGTAGGC |
| P2 | TCAGAAGATGCACCTTCTGCTGG |
| P3 | TGCTTCCGCTTCCTGAATGCTG |
| BMAL1 screen |  |
| Fw | TGGAGCACAGCTGCACTTAT |
| Rev | AAGTCCCAGCTGCACACAAG |
| qPCR analysis |  |
| *Gapdh-fw* | CATGGCCTTCCGTGTTCCTA |
| *Gapdh-rev* | CCTGCTTCACCACCTTCTTGA |
| *Tip60-fw* | GCCTGGACGGAAGCGGAAAT |
| *Tip60-rev* | CAATGTCGTGGCTCCGGT |
| *18S-fw* | AGTCCCTGCCCTTTGTACACA |
| *18S-rev* | GATCCGAGGGCCTCACTAAAC |
| *Actb-fw* | TTGTCCCCCCAACTTGATGT |
| *Actb-rev* | CCTGGCTGCCTCAACACCT |
| *36b4-fw* | AGATGCAGCAGATCCGCAT |
| *36b4-rev* | GTTCTTGCCCATCAGCACC |
| *c-Fos-fw* | TTCCTGGCAATAGCGTGTTC |
| *c-Fos-rev* | TTCAGACCACCTCGACAATG |
| *c-Jun-fw* | GAAGTGACGGACCGTTCTATGAC |
| *c-Jun-rev* | GGAGGAACGAGGCGTTGAG |
| ChIP analysis |  |
| Dbp -0.4-fw | ACACCCGCATCCGATAGC |
| Dbp -0.4-rev | CCACTTCGGGCCAATGAG |
| Dbp -0.2/Prom-fw | ACACAAGTTCAGCCCCTCAC |
| Dbp -0.2/Prom-rev | GGCAAGAACCAATCACGTCT |
| Dbp TSS-fw | AAGCTCCTTTCTTTGCGAGA |
| Dbp TSS-rev | TCAAGCAGCTGTCTCTTTGC |
| Dbp +0.2-fw | TCTGCAGAGCAGACTGGTTGA |
| Dbp +0.2-rev | GCGTGCAAACCTCCAGGAT |
| Dbp +0.8-fw | ATGCTCACACGGTGCAGACA |
| Dbp +0.8-rev | CTGCTCAGGCACATTCCTCAT |
| Dbp +2.4-fw | TGGGACGCCTGGGTACAC |
| Dbp +2.4-rev | GGGAATGTGCAGCACTGGTT |
| Dbp +4.4-fw | AAGAACAATGAAGCAGCCAAGAG |
| Dbp +4.4-rev | GGCAGCCCGCACAGATAT |
| Dbp +5.2-fw | GGCACCGGAGTAGGCAAGA |
| Dbp +5.2-rev | GCCTGGAATGTATGAGCTAGCA |
| Per1 -1.5-fw | CACCATGCCCAGCTTAGAA |
| Per1 -1.5-rev | CACCTTTGGAAGAGCAGTCA |
| Per1 -0.5-fw | GGCACCAGAAACCTCTTGTA |
| Per1 -0.5-rev | CCTTGACGACACTTACCCAATA |
| Per1 TSS-fw | AGCCGTCGAACTTGTGTTT |
| Per1 TSS-rev | TCTCCACAACCATTAGGCATTT |
| Per1 +0.5-fw | GGATTCTGAAAGGGAGGATAAGG |
| Per1 +0.5-rev | CAGAGCAGTCCTTTGGTAACT |
| Per1 +4.9-fw | CCGAGGCCTCAGAGTCCCA |
| Per1 +4.9-rev | GGCCATCTCTTACACCCCTT |
| Per1 +14.3-fw | TGGACAGACCACTTCAGCAG |
| Per1 +14.3-rev | GAAGTTCTCAGCCCCATTTG |
| Per1 Prom-fw | GGCATTTGCGTCACTGATTTAG |
| Per1 Prom-rec | GATTGGACATCTGACCGGAAG |
| Nr1d1 -2.1-fw | AGCTAGTGAGAAGAGGGAACTA |
| Nr1d1 -2.1-rev | GCTTCATACCTCACACAGACTAC |
| Nr1d1 -0.5-fw | ACAAGCTGGGAGGAGAGAT |
| Nr1d1 -0.5-rev | GGGTTAGGGTGAAGAAGAGAAAG |
| Nr1d1 TSS/Prom-fw | AGGCACACTCCACCTACATTGTCA |
| Nr1d1 TSS/Prom-rev | TGGAGCAGGTACCATGTGATTCCA |
| Nr1d1 +0.1-fw | CGCTCGGAACTGTGAGTAG |
| Nr1d1 +0.1-rev | GGCATTTGTTCTGCAGCTAAT |
| Nr1d1 +2.0-fw | TCATGCCCTCTTTCAGGATT |
| Nr1d1 +2.0-rev | TTACCCGGCTATGGTTTCAC |
| Nr1d1 +6.8-fw | CTCCTTCTCCTTTCCCGTTG |
| Nr1d1 +6.8-rev | CCTGAGCTCCTCCTCTGACA |
| Control-fw | TGCTAGATGCTGCGGAAGAACTGA |
| Control-rev | TCTCTATGCTCCCAGCCAAGGTAT |
